# The Ciliary Protein Cystin Forms a Regulatory Complex with Necdin to Modulate *Myc* Expression

**DOI:** 10.1371/journal.pone.0083062

**Published:** 2013-12-11

**Authors:** Maoqing Wu, Chaozhe Yang, Binli Tao, Su Bu, Lisa M. Guay-Woodford

**Affiliations:** Research Division, Department of Genetics, University of Alabama at Birmingham, Birmingham, Alabama, United States of America; University of Massachusetts Medical, United States of America

## Abstract

Cystin is a novel cilia-associated protein that is disrupted in the *cpk* mouse, a well-characterized mouse model of autosomal recessive polycystic kidney disease (ARPKD). Interestingly, overexpression of the *Myc* gene is evident in animal models of ARPKD and is thought to contribute to the renal cystic phenotype. Using a yeast two-hybrid approach, the growth suppressor protein necdin, known to modulate *Myc* expression, was found as an interacting partner of cystin. Deletion mapping demonstrated that the C-terminus of cystin and both termini of necdin are required for their mutual interaction. Speculating that these two proteins may function to regulate gene expression, we developed a luciferase reporter assay and observed that necdin strongly activated the *Myc* P1 promoter, and cystin did so more modestly. Interestingly, the necdin effect was significantly abrogated when cystin was co-transfected. Chromatin immunoprecipitation and electrophoretic mobility shift assays revealed a physical interaction with both necdin and cystin and the *Myc* P1 promoter, as well as between these proteins. The data suggest that these proteins likely function in a regulatory complex. Thus, we speculate that *Myc* overexpression in the *cpk* kidney results from the dysregulation of the cystin-necdin regulatory complex and c-Myc, in turn, contributes to cystogenesis in the *cpk* mouse.

## Introduction

Polycystic kidney disease (PKD) is a group of genetic disorders characterized by the progressive development of renal cysts, ultimately leading to end-stage renal disease [1]. PKD is further defined by inheritance as either autosomal dominant polycystic kidney disease (ADPKD) or autosomal recessive polycystic kidney disease (ARPKD). ADPKD occurs with an estimated prevalence of 1∶400 to 1∶1,000 live births. Symptoms may manifest at any time in life and are characterized by cyst formation in ductal organs (particularly the kidney and liver), in addition adults may present with gastrointestinal, cardiovascular, and musculoskeletal abnormalities. Overall, ADPKD accounts for ∼8% of cases of end-stage renal disease [2]–[5]. Mutations in one of two genomic loci, *PKD1* (encoding polycystin 1, PC-1) or *PKD2* (encoding polycystin 2, PC-2), are associated with ADPKD phenotypes that are similar in clinical presentation [6], [7]. Approximately 85% of ADPKD cases are caused by mutations in the *PKD1* gene, and the remaining 15% can be attributed to mutations in the *PKD2* gene [8]–[12]. While the functions of each protein are not fully understood, it has been demonstrated *in vitro* that these proteins associate in the membrane of the primary apical cilia as a mechano-sensitive complex that regulates calcium signaling [13], [14].

In contrast to the dominant form of PKD, ARPKD is much less prevalent and generally more severe. ARPKD occurs once in 20,000 live births and is characterized by cystic kidneys and congenital hepatic fibrosis with a high rate of mortality in affected newborns [12], [15]. In a subset of patients, the disease phenotype predominantly involves portal hypertension or cholangitis [15], [16]. Genetic analysis has shown that mutations in *PKHD1*, which encodes the fibrocystin/polyductin (FPC) protein, cause ARPKD [17]–[19]. Initial studies suggested that disease severity correlated with the type of mutation [12], [17]–[21], however, this association has not been substantiated [22], [23]. Indeed, mice with mutations in *Pkhd1* do not display phenotypes similar to ARPKD. In sum, the molecular functions of FPC are poorly defined. However, like many of the other ciliary proteins, FPC appears to play a role in the structural integrity of cilia, as well as facilitating ductal epithelial differentiation, by participating in multiple cellular events, such as proliferation, secretion, apoptosis, and terminal differentiation [24]–[28]. It is also increasingly evident that FPC interacts with PC-2 at the cilium [29]–[31].

Among the mouse models for ARPKD, the congenital polycystic kidney (*cpk*) mouse with a mutation in cystin, was the first to be described and remains the most extensively characterized [32]–[34]. The renal phenotype of the *cpk* mouse is strikingly similar to human ARPKD despite being genetically distinct from the human disorder [35], [36]. Our group has demonstrated that the gene *Cys1* encodes cystin, a novel lipid-microdomain associated protein that co-localizes with PC-1, PC-2, and Ift88 in the primary apical cilia of renal epithelia cells [37]–[39]. Recent studies have demonstrated that *Cys1* is a target of the transcriptional complex that is regulated by the hepatic nuclear factor (HNF) transcription factors [40]–[42]. However, any role cystin plays in human disease or cystogenesis remains unclear.

In the present study, we performed yeast two-hybrid (Y2H) screening and identified putative interacting partners of cystin. Experiments demonstrated that the growth suppressor and transcriptional regulator necdin interacts with cystin. Necdin has been shown to interact with several cystogenic related proteins and may play a role in PKD. Using multiple approaches, such as luciferase reporter assays, chromatin immunoprecipitation (ChIP), and electrophoretic mobility shift assays (EMSA), we determined that cystin forms a regulatory complex with necdin and modulates the expression of the c-Myc gene, *Myc*. Interestingly, overexpression of *Myc* is well documented in *Cys1^cpk/cpk^* kidneys [43], [44]. We speculate that the loss of cystin function or the disruption of this regulatory complex results in the overexpression of *Myc*, which alters downstream targets and contributes to cystogenesis in the *cpk* mouse model of ARPKD.

## Results

### Yeast two-hybrid screening

In previous studies, we showed that cystin, the protein product of *Cys1* that is mutated in the *cpk* mouse, is localized within the ciliary membrane of kidney epithelial cells. Interestingly, a myristoylation mutant termed cystin_G2A_, was shown to accumulate in the nucleus [38]. This suggested that cystin exits the cilium and may function in the nucleus in certain physiological conditions. Thus, we sought to identify cystin interacting partners by yeast two-hybrid screening. We cloned full-length mouse cystin and performed two rounds of Y2H screening using an embryonic day 17 mouse cDNA library (Table 1). Using highly stringent selection, we identified several putative interacting partners (data not shown). Necdin was of particular interest because it is known to interact with p53 and participate in gene regulation. In order to eliminate any false positives, and to validate the results, we reversed the bait and prey pairs and re-screened the interaction (Table 2). This further confirmed that in yeast, the interaction between cystin and necdin was genuine.

**Table 1:** Y2H validation of the necdin and cystin interaction.

Vector Pairs	SD/-Ade-His-Leu-Trp/X-gal
pGBKT7-p53+ pGADT7-LgT	+
pGBKT7-Lam+ pGADT7-LgT	–
pGBKT7+ pACT2-necdin	–
pGBKT7-cystin+pACT2-necdin	+

p53+LgT and Lam+LgT were used as positive and negative controls, respectively for protein interaction. Necdin was identified from a cDNA library as a full-length cystin binding partner. LgT: large T-antigen; Lam: human lamin C. pGBKT7 is a bait vector containing the GAL4 DNA binding domain; pGADT7 and pACT2 are prey vectors containing the GAL4 activation domain.

**Table 2:** Vector switch to eliminate false positive clones.

	SD/-Ade-His-Leu-Trp+3-AT (mM)						SD/-Leu-Trp
	0	2.5	5	7.5	10	15	
pGBKT7-p53+pGADT7-lgT	+	+	+	+	+	+	+
pGBKT7-Lam+pGADT7-lgT	–	–	–	–	–	–	+
pGBKT7+pGADT7-cystin	–	–	–	–	–	–	+
pGBKT7-necdin+pGADT7	–	–	–	–	–	–	+
pGBKT7-necdin+pGADT7-cystin	+	+	+	+	+	+	+

p53+LgT and Lam+LgT were used as positive and negative controls, respectively. Necdin was switched from the prey vector (pGADT7) to the bait vector (pGBKT7). The new constructs were re-tested and their interactions were validated. 3-AT: 3-amino-1,2,4-triazole, a competitive inhibitor of the His3 protein.

**Figure 1:**
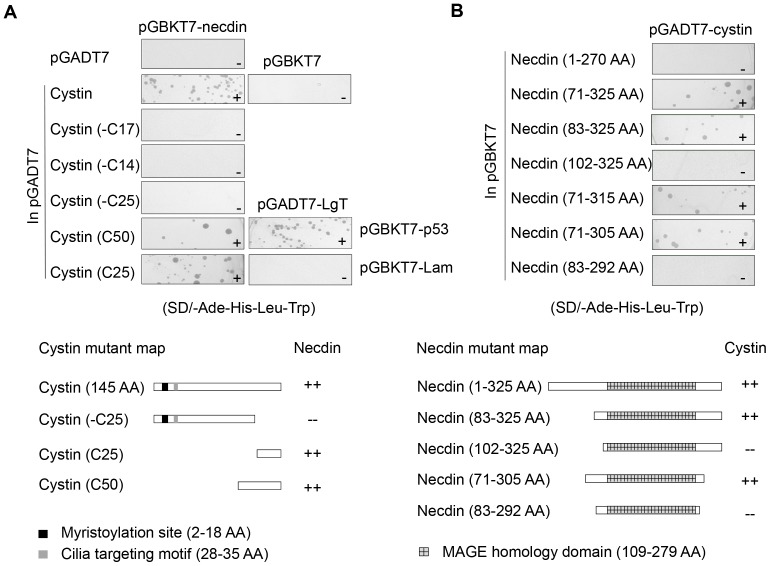
An interaction between cystin and necdin was identified in a yeast two-hybrid screen. (**A**) After the initial unbiased screen full-length cystin was cloned into a prey vector, while necdin was cloned into a bait vector. Both were co-transformed and spread onto control SD/-Leu-Trp (data not shown) and selective SD/-Ade-His-Leu-Trp plates. A series of deleted cystin constructs were co-transformed with full-length necdin to isolate the necdin-interacting domain. Y2H positive (pGADT7-LgT+pGBKT7-p53) and negative controls (pGADT7-LgT+pGBKT7-Lam) are also shown. A scheme of the interactions between the versions of cystin and necdin are shown at the lower panel. The C-terminal 25 aa of cystin are required for its interaction with necdin. (**B**) A series of necdin constructs (prey) were co-transformed along with full-length cystin (bait) to determine the cystin-interacting domain. A scheme of the interaction between the versions of necdin and cystin are shown in the lower panel. Necdins cystin interaction domain lies within aa 71 - 305.

In order to define the interaction domains in both cystin and necdin, we made a series of deletion constructs (lower panel of Figure 1A and 1B), and evaluated their interaction in the same Y2H system. The data showed that the deletion of C-terminal 25 amino acids (aa) of cystin, cystin-C25, abolished its ability to interact with necdin. Interestingly, the carboxy terminal fragment of cystin, alone, is enough to bind necdin (Figure 1A, upper panel). Regarding necdin, there appears to be two cystin interaction domains on either side of the melanoma antigen (MAGE) domain within aa 71 to 305 (Figure 1B, lower panel). Combined with the Y2H results, we conclude that the cystin and necdin specifically interact.

### Cystin interacts with necdin

**Figure 2:**
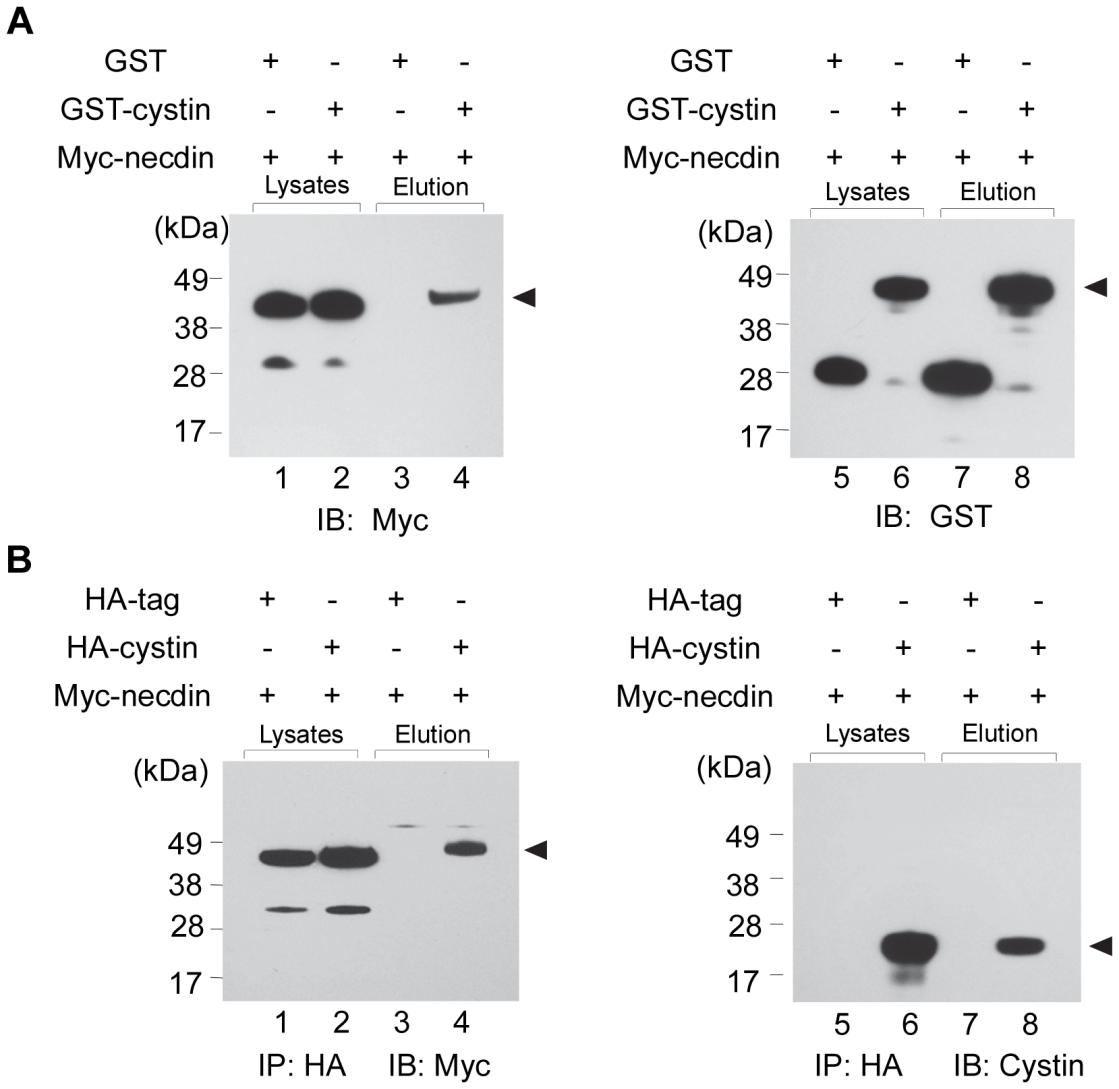
The interaction between cystin and necdin was further verified by GST pull-down and co-immunoprecipitation. (**A**) GST pull-down was used to verify the interaction between cystin and necdin. pCMV-GST-cystin and pCMV-myc-necdin were co-transfected into COS-7 cells. The precipitated samples were run on a PAGE gel and probed with myc (lanes 1-4) and GST antibodies (lanes 5-8). Whole cell lysate was used to assess protein expression (lane 1-2 and 5-6). Compared to the negative control (lane 3), the specific interaction between cystin and necdin is clear (lane 4, arrowhead). (**B**) Co-IP was used to verify the interaction between cystin and necdin. pCMV-HA-cystin and pCMV-myc-necdin were co-transfected into COS-7 cells. The protein complex of HA-cystin and myc-necdin in the cell lysate was precipitated using the HA tag, and the samples were probed with myc (lanes 1-4) and cystin antibodies (lanes 5-8), respectively. Whole cell lysate was used to control for protein expression (lane 1-2 and 5-6). Compared with the negative control (lane 3), a specific band indicates a physical interaction between cystin and necdin (lane 4, arrowhead).

Following the Y2H screening, we used GST pull-downs and co-immunoprecipitation to verify the interaction between cystin and necdin in a mammalian system. We cloned full-length cystin into a GST vector and full-length necdin into a myc vector. These constructs were co-transfected into COS-7 cells and grown for 48 hours before lysis. Under the same binding and washing conditions, myc-necdin was efficiently pulled-down by GST-cystin (Figure 2A, left panel, arrowhead), while GST alone did not (Figure 2A, lane 3). The lysates and elution were probed with GST as both a transfection and loading control (Figure 2A, lanes 5-8).

Co-immunoprecipitation experiments were performed for further validation. Full-length cystin and necdin were cloned into an HA and myc vector, respectively, and co-transfected into COS-7 cells. The cell lysates were immunoprecipitated with an HA antibody and probed with the myc antibody. As expected, HA-cystin interacted strongly with myc-necdin (Figure 2B, lane 4) while HA alone did not (Figure 2B, lane 3). The lysates and elutions were also probed with a cystin antibody, to act as both a transfection and loading control (Figure 2B, right panel). Taken together, the data show that cystin interacts with necdin in yeast as demonstrated by Y2H, and in mammalian cells by GST and co-immunoprecipitation.

### Necdin and cystin are expressed in mouse kidney epithelial cells

**Figure 3:**
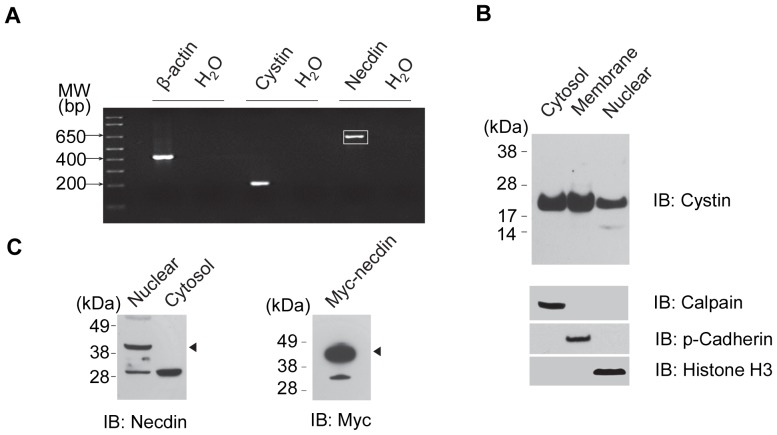
Endogenous cystin and necdin were expressed in mIMCD-3 cells. (**A**) RT-PCR was performed to detect the expression of cystin and necdin in mIMCD-3 cells. Total RNA was isolated from confluent mIMCD-3 cells and equal amounts of cDNA were used as a template for RT-PCR. Primer pairs for cystin and necdin were designed to amplify 207 bp and 632 bp target bands, respectively. β-actin was used as a positive control; water was used as a negative control. The PCR products were excised from the gel, purified, and verified by sequencing (data not shown). (**B**) Western blot analysis of endogenous cystin in confluent mIMCD-3 cells. The cells were fractioned to cytoplasmic, membrane, and nuclear fractions. Analysis of control proteins for the different fractions were shown in the lower panel. Endogenous cystin migrates at 25 kDa and accumulated in the membrane fraction. **(C)** Western blot analysis of endogenous necdin in confluent mIMCD-3 cells. Endogenous necdin was detected in the nuclear fraction and migrated at 40 kD when probed with a necdin N-terminal antibody (N20, arrowhead). Western blot analysis of Myc-necdin in mIMCD-3 cells using a myc antibody also reveals a band at 40 kD (right). The band at ∼30 kDa is an unidentified artifact of the antibody used [103].

Following confirmation that necdin physically interacts with cystin *in vitro*, we explored whether necdin and cystin were expressed in the same cell types, specifically mIMCD-3 (mouse inner medulla collecting ducts) cells. In order to evaluate the expression of the endogenous cystin and necdin at the transcript level, we isolated total RNA from confluent mIMCD-3 cells and performed RT-PCR. The specific products of cystin (207 bp) and necdin (632 bp) (Figure 3A) were amplified and verified by nucleotide sequence analysis (data not shown). In addition, we assessed endogenous necdin and cystin at the protein level. Cellular fractions were prepared from confluent mIMCD-3 cells grown in 10 cm plates. Western blots with our cystin antibody (70053 [37]) showed that cystin is highly expressed in confluent mIMCD-3 cells. The target band of cystin, ∼25 kD, was specifically detected in the cytoplasm, membrane, and nucleus (Figure 3B). Necdin was only detectable in the nucleus and not in the cytoplasm under these conditions (Figure 3C, left panel, arrowhead) and is robustly expressed, exogenously, in whole cell lysates (Figure 3C, right panel).

### Cystin has a functional nuclear localization signal (NLS)

**Figure 4:**
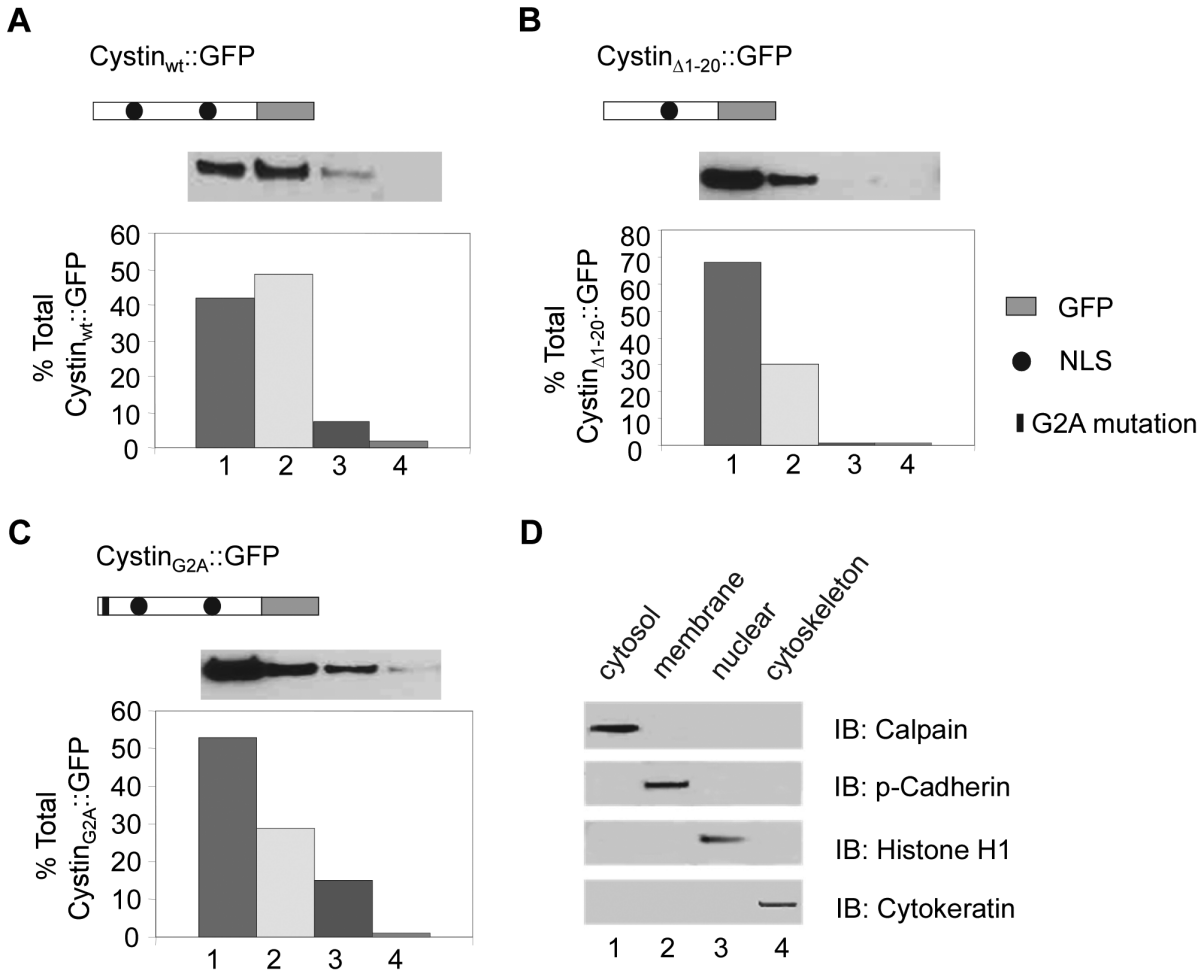
Cystin has a functional nuclear localization signal at the N-terminus. The subcellular localization of cystin and its variants was determined by immunoblotting. The wild-type cystin_wt_, the N-terminal deletion mutant cystin_▵1-20_, and the site-directed mutant cystin_G2A_, each tagged with GFP were stably-transfected in mIMCD-3 cells. (**A**) The distribution of wild-type cystin_wt_::GFP in the four fractions prepared from mIMCD-3 cells is shown by western blot (above) and using densitometry (below). (**B**) The distribution for cystin_▵1-20_::GFP is similarly shown by western and densitometry. (**C**) The distribution of the site-directed mutant cystin_G2A_::GFP is shown in the four fractions of mIMCD-3 cells using western blot analysis and densitometry. (**D**) To demonstrate the reliability of the fractionation protocol, wild type mIMCD-3 cells were fractionated (n = 5 experiments) and 10 µg of each fraction was analyzed by western blotting with fraction specific marker as indicated. The fractions are cytosol (1), membrane (2), nuclear (3) and cytoskeleton (4).

Bioinformatic analysis predicted that cystin contains two nuclear localization signals (termed NLS1 and NLS2, Figure 4A [38]). Using a subcellular fractionation approach, we examined whether NLS1 was functional using modified versions of cystin. First, we performed subcellular fractionation (n = 5 experiments) in wild type mIMCD-3 cells to assess the reproducibility of the technique and performed western blotting to confirm that there was no contamination among fractions (Figure 4D). Stably-transfected cell lines were then processed in parallel to determine the subcellular localization of the cystin::GFP variants. For each stably-transfected mIMCD-3 cell line, western blotting was performed after fractionation and the relative levels of cystin therein were assessed by densitometry. The bars represent the relative amount of cystin in each fraction (total sums to 100%). In Figure 4A, the fractions are cytosol (1), membrane (2), nuclear (3) and cytoskeleton (4). Cystin_wt_::GFP was associated with the membrane but was also detected in the cytoplasm. However, less than 10% was found in the nucleus (Figure 4A). The cystin_▵1-20_::GFP construct lacking both the NLS1 and myristoylation domains was observed largely in the cytoplasm and was strikingly decreased in the nuclear fraction (Figure 4B). In contrast, the cystin_G2A_::GFP protein was not dramatically increased in the cytoplasm, despite the fact that it was unable to associate with the membrane. However this variant was substantially increased in the nucleus, likely as a result of a functional NLS1 (Figure 4C). We speculate that under specific physiological conditions, cystin can cycle out of the ciliary membrane and translocate into the nucleus to regulate gene expression.

### Cystin is co-localized with necdin in mIMCD-3 cells

**Figure 5:**
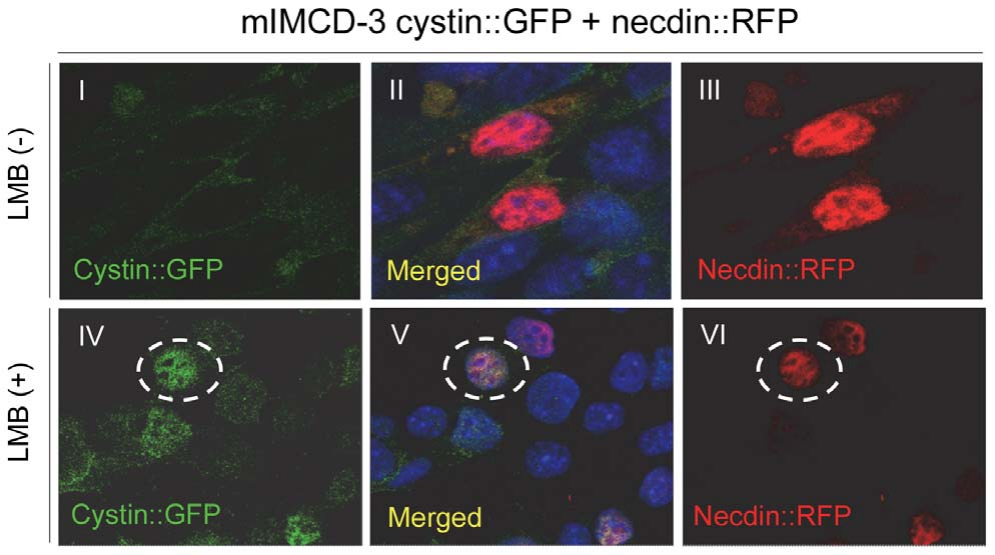
Exogenous cystin and necdin were co-localized in mIMCD-3 cells. A stable cell line transfected with both cystin::GFP and necdin::RFP, was established in mIMCD-3 cells. Under identical conditions, the co-localization of cystin and necdin was determined with and without leptomycin B (LMB) treatment. As shown by epifluorescence, in confluent mIMCD-3 cells, cystin::GFP was primarily retained in the cytoplasm, while necdin::RFP was mainly distributed in the nucleus (I-III). Cystin and necdin only minimally co-localize in the cytoplasm. After LMB treatment (80 nM, 5 hrs lower panels, IV-VI), cystin::GFP was retained in the nucleus and co-localized with necdin (V, white dashed oval).

In order to evaluate whether cystin and necdin co-localize in mIMCD-3 cells, we created a cystin::GFP and necdin::RFP (red mono) stable cell line. We found necdin in the cytoplasm and largely in the nuclei of confluent mIMCD-3 cells (Figure 5, II and V). Conversely, most of the cystin::GFP was found in the cytoplasm while much less of it was in the nucleus (Figure 5, I). Based on our previous studies, we predict that cystin can traffic between the nucleus and cytoplasm [38]. Thus, a strategy to verify this hypothesis was to block nuclear export, thereby retaining any cystin that did enter the nucleus. We used the nuclear export inhibitor leptomycin B, upon optimization we found that 80 nM was effective at causing cystin_G2A_::GFP nuclear retention (Figure 5, IV). Consistent with our hypothesis, cystin visibly accumulated in the nucleus and co-localized with necdin (Figure 5, V). This observation suggests that cystin can translocate from the cilia and/or cytoplasm into the nucleus and is in a position to complex with necdin to function therein.

### Cystin antagonizes necdin to modulate *Myc* P1 promoter activity

**Figure 6:**
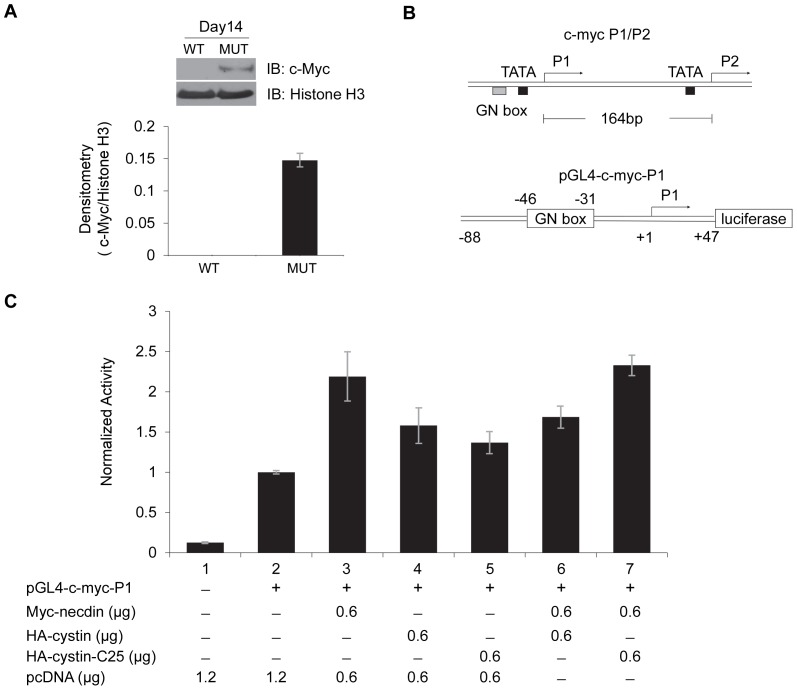
The activation effect of necdin on *Myc* P1 promoter activity is antagonized by cystin. (**A**) Immunoblot analysis of c-myc expression in total kidney lysates from 14 day-old *Cys1^cpk/cpk^* (MUT) and wild-type (WT) mice, densitometry shown below. (**B**) Schematic structures of the *Myc* P1 and P2 genomic promoter (upper) and the *Myc* P1 luciferase construct (lower) where *Myc* P1 DNA from –88 to +47 was cloned into pGL4.22 vector. (**C**) Luciferase analysis of the effect of necdin and cystin on *Myc* P1 activity. Basal P1 activity (lane 2) is elevated ∼1.5 fold by necdin (lane 3) and to a lesser extent by cystin alone (lane 4). However, cystin abrogates the ability of necdin to activate the P1 element when co-transfected (lane 6), as long as the necdin interacting domain remains intact [i.e. the –C25 mutant does not inhibit necdin (lane 7)]. Error bars represent the 95% confidence interval for the SEM (p<0.05).

Cystin is a small protein that has no readily identifiable DNA binding domain as determined by bioinformatic analysis, suggesting that for cystin to regulate gene expression, it would need to interact with other transcriptional regulatory proteins, like necdin. Therefore, we sought to determine whether these two proteins interact to modulate *Myc* expression. It has been confirmed that c-Myc is over expressed in *cpk* kidneys [43], [44] and that necdin activates the *Myc* expression through binding to its GN box in the promoter [45]. Western blot data demonstrates that c-Myc is highly expressed in the 14 day-old mutant *cpk* mouse compared to a wild-type littermate (Figure 6A). The expression of *Myc* is regulated by two major promoters, P1 and P2, with transcription initiation sites 164 base pairs apart [46] (Figure 6B, upper map). Interestingly, most of c-Myc overexpression results from dysregulation of the P1 promoter element [47]. Therefore, we cloned the *Myc* P1 promoter, ranging from bp -88 to +47 (Figure 6B, lower map), into a luciferase reporter vector and performed dual-luciferase reporter assays (Figure 6C). Our data showed that in mIMCD-3 cells, necdin activated the *Myc* P1 promoter (Figure 6C, lane 3), consistent with previously published data [45]. We then tested the effect of cystin on *Myc* P1 promoter activity in mIMCD-3 cells. Alone, cystin was able to significantly activate the *Myc* P1 promoter (Figure 6C, lane 4), and intriguingly, the necdin-interaction deficient version of cystin (-C25 construct) also activated the P1 promoter (Figure 6C, lane 5). The magnitude of stimulation by necdin (1.5 fold) was greater than that of cystin (0.5 fold). Importantly however, when in co-transfection assays, cystin antagonized the activity of necdin, resulting in activation levels similar to that of cystin alone (Figure 6C, lane 6). Further, we found that the cystin modulation of necdin-associated *Myc* P1 promoter activity was dependent on the necdin-binding domain in the cystin C-terminus. Necdin activation of the *Myc* P1 promoter was not inhibited by the –C25 construct (Figure 6C, lane 7). These data demonstrate that cystin and necdin can both activate the *Myc* P1 promoter (albeit necdin has a more potent effect), and that cystin antagonizes the stimulatory effect of necdin through a direct protein-protein interaction. Taken together, our data indicate that cystin is a key modulator of c-Myc expression.

### Both cystin and necdin bind to *Myc* P1 DNA

**Figure 7:**
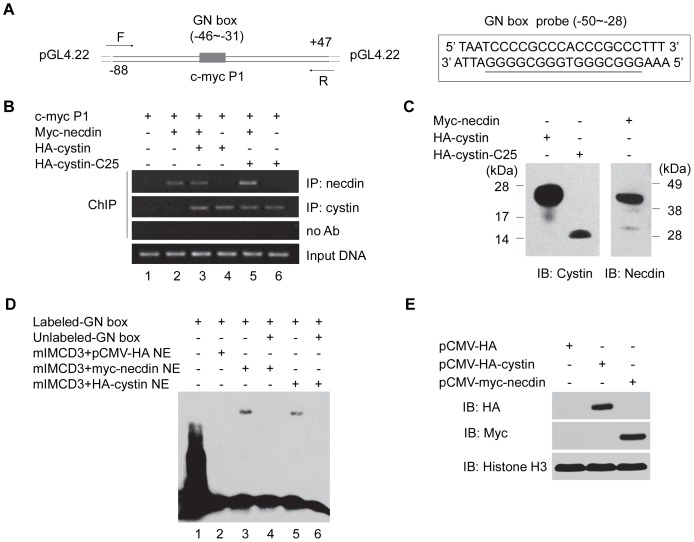
Both necdin and cystin bind to *Myc* P1 promoter DNA. (**A**) Schematic representation of the *Myc* P1 promoter (–88 ∼ +47) fragment in the pGL4.22 luciferase vector used for chromatin immunoprecipitation (ChIP). The ChIP primers (F and R) spanned the promoter region. The right panel showes the 23-bp EMSA probe, containing the *Myc* P1 GN-box (underlined). (**B**) ChIP was performed to identify the interaction of necdin, cystin, and cystin-C25 with the *Myc* P1 promoter fragment. After sonication, the protein-DNA complexes were immunoprecipitated either with the anti-necdin (N143) or cystin (70053) antibody. **(C)** Western blot analysis confirmed that each of the constructs were highly expressed in the ChIP experiments. HA-cystin/HA-cystin-C25 were detected with the anti-cystin antibodies (70053) and myc-necdin was detected with an anti-necdin antibody (N143). (**D**) EMSA was performed to analyze the binding of necdin and cystin to the *Myc* P1 DNA fragment. The biotin-labeled probe (A right panel) containing the *Myc* P1 GN box was incubated with nuclear extracts from mIMCD-3 cells transfected with wild-type cystin and necdin. Both cystin and necdin bound to the *Myc* P1 GN box (lanes 3, 5) and this binding was efficiently abolished by unlabeled probe. (**E**) Immunoblotting analysis of the EMSA samples indicated robust expression of HA-cystin and myc-necdin in nuclear extracts, histone H3 was used as a loading control.

Our reporter assay data in mIMCD-3 cells suggested that necdin and cystin function together to regulate the *Myc* P1 promoter, but whether this was through directly binding the DNA is unknown. ChIP experiments were used to assess the interaction between the *Myc* P1 DNA and the necdin and cystin proteins. We co-transfected *Myc* P1, myc-necdin, HA-cystin, or HA-cystin-C25 in various combinations into COS-7 cells. Western blots confirmed that myc-necdin, HA-cystin, and HA-cystin-C25 were all highly expressed in COS-7 cells, and the antibodies used for IP were specific to the proteins expressed (Figure 7C). After immunoprecipitation with antibodies against cystin or necdin, we performed PCR with specific primers spanning the full-length *Myc* P1 promoter to amplify any bound *Myc* P1 DNA (Figure 7A). The results indicate that necdin (Figure 7B, lanes 2, 3, 5), cystin (Figure 7B, lanes 3, 4), as well as cystin (C-25) (Figure 7B, lanes 5, 6) immunoprecipitated with the *Myc* P1 DNA fragment. This data, together with the luciferase data (Figure 6C), further suggests that cystin can bind the *Myc* P1 promoter without necdin.

In order to confirm that cystin and necdin can bind to the *Myc* P1 promoter DNA, we performed an electrophoretic mobility shift assay (EMSA). To ensure that the samples used for the EMSA experiments were biologically relevant, we used nuclear extracts from mIMCD-3 cells that had been transfected with myc-necdin, HA-cystin, or an empty vector, respectively. We used 5′ biotin-labeled oligonucleotides that contained the *Myc* P1 GN box as a probe (–50∼–28) (Figure 7A, right side map). The shifted DNA-protein complexes were readily observed in the mixtures containing transfected necdin or cystin and the labeled GN box probe (Figure 7D, lanes 3, 5). As controls, the empty vector mIMCD-3 extract did not show any shifted band, and unlabeled probe was able to compete and abolish the interaction (Figure 7D, lanes 2, 4, 6). Expression of cystin or necdin in the nuclear input was confirmed (Figure 7E). Combined with our reporter assay, both the ChIP and EMSA data confirm that both cystin and necdin interact with the *Myc* P1 promoter fragment and likely function as a regulatory complex modulating *Myc* expression.

## Discussion

In this study, we employed a Y2H approach using a full-length cystin construct to screen a mouse embryo cDNA library for cystin-interacting partners under stringent selection conditions (SD/-Ade/-His/-Leu/-Trp/X-gal). Necdin was among the putative interacting partners identified in this screen. Subsequently, the physical interaction of these two proteins was confirmed by GST pull-down and co-immunoprecipitation, and is supported by immunofluorescent experiments showing colocalization of the proteins at the subcellular level. From the perspective of renal cystic epithelia biology, necdin is an interesting cystin binding partner. Necdin was first identified in mouse stem cells of embryonic carcinoma origin [48] and has been detected in the nucleus of differentiated neuronal cells, but not in undifferentiated cells [49]–[54]. Further, necdin has been identified as a maternally imprinted gene and is implicated as a disease-causing gene associated with Prader-Willi syndrome [49], [55], [56]. Necdin is a DNA-binding protein [57] that binds to the GN box of the *Myc* P1 promoter and regulates its activity [45]. The N-terminus of necdin is proline-rich, which is a typical feature of trans-activation/repression domains of transcription factors [58], [59]. In addition, necdin has more recently been linked to p53 signaling, histone acetylation, calcium signaling, and interacts with multiple transcription factors (e.g. Dlx5, SIM1, and Msx2) [51], [53], [60]–[63].

To date, a number of interacting partners of necdin have been identified, some of which are renal cystic disease-related proteins, including Bbs4, HIF1α, p53, and E2F1 [50], [61], [64], [65]. Bbs4 is encoded by one of the causative genes of Bardet-Biedl syndrome (BBS), an inherited ciliopathy characterized by renal cystic disease, obesity, polydactyly, and diverse neuropsychiatric symptoms [66], [67]. Further, deficiencies in p53, a well-known tumor suppressor, are associated with a renal cystic phenotype during embryonic development [68]. Necdin also appears to mediate the degradation of hypoxia-inducible factor 1 alpha (HIF1α), whose overexpression is involved in renal cyst formation [69], [70]. In post-mitotic neurons, necdin acts as an anti-apoptotic factor by repressing E2F1-dependent *Cdc2* transcription, thus stabilizing neuronal terminal differentiation [71]. While necdin is associated with multiple renal cystic disease-related genes, a kidney phenotype in necdin mutant mouse models has not been well studied [63].

The proto-oncogene *c-Myc* was first discovered in Burkitt's lymphoma patients [72]. It is a basic Helix-Loop-Helix/Leucine Zipper transcription factor that binds to enhancer box sequences (E-box: CACGTG) and recruit histone acetyltransferases to transcriptionally regulate the expression of 15% of all genes, including genes involved in growth, proliferation, cell cycle progression, apoptosis, and differentiation [73]. Dysregulated expression of c-Myc contributes to the genesis of multiple types of human tumors [74] and targeting *Myc* overexpression in stenosis is the focus of current clinical trials (http://clinicaltrials.gov/ct2/show/study/NCT00777842; National Institutes of Health. Bethesda, MD) [75]. The mechanisms that govern *Myc* transcription are complex and involve multiple promoters (P0, P1, P2, and P3) and start sites. As noted above, the P1 and P2 promoters are the predominant *Myc* regulatory elements and most ectopic *Myc* expression results from dysregulation at the P1 promoter.

Overexpression of *Myc,* a common feature of PKD, was first described in *cpk* kidneys [43], [44]. Presently, it is thought that c-Myc overexpression is a hallmark of PKD and cystogenesis in general [76]–[86]. Mouse and pig transgenic models designed to overexpress c-Myc manifest a PKD-like renal phenotype [87], [88]. Of additional note, a newly identified variant myc-Nick is associated with microtubule hyperacetylation [89], a characteristic of renal cystic epithelia [90]. Further, alterations in c-Myc expression have led to speculation about the possible role for cilia and cilia-related genes in the modulation of Wnt and Hippo signaling [81], [91]–[93]. Interestingly, the necdin-interacting partners mentioned above, including HIF1α, p53, and E2F1 have also been shown to be targets of c-Myc [94]–[97]. In addition, other renal cystic disease-related genes, adenomatosis polyposis coli (APC) and Von Hippel-Lindau (VHL) are also directly targeted by c-Myc [94], [98], [99]. Perhaps most importantly, a study by Ricker et al. used *Myc* antisense oligonucleotides to successfully attenuate the ARPKD phenotype in *cpk* mice [100]. These reports support the hypothesis that upregulation of *Myc* is a significant cystogenic factor in *cpk* kidneys.

In this study we demonstrate that cystin and necdin interact as a complex with the P1 promoter to regulate *Myc* expression. Our data show that necdin enhances *Myc* promoter activity and that cystin antagonizes this effect. These data are consistent with *in vivo* observations that *Myc* is overexpressed in *cpk* kidneys and anti-*Myc* nucleotides mitigate the cystic kidney phenotype [100]. Importantly, we noted that there was significant variability in the luciferase assays when veteran mIMCD-3 cells (>50 passages) were used, suggesting that necdin and cystin have dynamic effects on gene expression that depends at least in part on the age of the cell line. Using low passage (<10) mIMCD-3 cells (American Type Culture Collection), our experimental data had much more limited variability and strong reproducibility. Since both the N- and C-termini of necdin are required for cystin interaction (Figure 1B, lower panel) and cystin C-25 does not inhibit the stimulatory effect of necdin on the P1 promoter, we speculate that the counteracting effect of cystin on necdin is likely dependent on the interaction of these two proteins, which in turn impacts their ability to bind DNA, transactivators, or other transcription factors.

Both cystin and necdin are expressed in the mIMCD-3 renal cell line, as detected by RT-PCR and western blot. To our knowledge, this is the first report of necdin expression in renal epithelial cells. It has been reported that necdin is localized in the nucleus, centrosome, and cytoplasm [50], [51], [101]. This distribution pattern resembles that of cystin (and other cystoproteins), although there is no evidence that necdin localizes to cilia. Thus, these two proteins may play a role in the regulation of cell cycle and/or centrosome duplication directly or indirectly through gene regulation. This report adds to a growing dataset of ciliary proteins with roles in mammalian gene expression (e.g. PC-1 and FPC) [29], [46], [102].

In summary, our data indicate that cystin and necdin independently bind to the *Myc* promoter, but their direct interaction is required to properly regulate *Myc* expression. We propose that *Myc* overexpression in the *cpk* mouse results directly from the disruption of this cystin-necdin regulatory complex, which allows necdin to aberrantly drive *Myc* expression. Based on our data, and the observations of others, that *Myc* overexpression is a common signature in cystic epithelia, we speculate that other cystoproteins and/or their downstream effectors may be targets of the cystin-necdin transcriptional regulatory complex.

## Materials and Methods

### Yeast two-hybrid screening

Cystin (full ORF, cloned into pGBKT7, the bait vector), was used to screen the embryonic day 17 mouse embryo cDNA library that was transformed into the Y187 yeast strain. The co-transformants surviving on SD/-Ade/-His/-Leu/-Trp/X-gal selection plates were positive candidates. To eliminate the false positives, cystin was switched into the pGADT7 prey vector and rescreened against the other putative interacting partners in the pGBKT7 vector. Yeast Transformation System 2 (Clontech) and the MATCHMAKER GAL4 Two-Hybrid System 3 (Clontech) were used.

### GST pull-down assay

GST pull-downs were performed per the ProFound Pull-Down GST Protein Interaction kit (Pierce). Both the cystin and necdin were cloned into the pCMV-GST vector (eukaryotic GST fusion vector) and the pCMV-myc vector, respectively. Briefly, 20 µg of plasmid DNA were co-transfected into COS-7 cells in 10 cm plates at 90% confluence with 30 µl Lipofectamine 2000 (Invitrogen). After ∼48 hrs cell growth, the cells were washed and lysed in 0.8 ml ProFound cell lysis buffer with proteinase inhibitor (complete Mini, EDTA-free, Roche), and incubated at 4°C for 30 min. About 0.7 ml of supernatant was collected after centrifugation. The cell lysates were incubated with 50 µl immobilized glutathione beads (50% slurry, 25 µl bed volumes) overnight on a rocking platform at 4 °C. After incubation, the samples were centrifuged at 1300 g for 30 sec to 1 min to collect GST protein complexes. The GST-glutathione beads were vigorously washed with cold cell lysis buffer 5–6 times and centrifuged. Finally, the protein/bead complex was eluted in ∼200 µl 100 mM Glutathione in cell lysis buffer. The purified proteins were subjected to western blot analysis with anti-GST antibody (Santa Cruz #80998), anti-Myc antibody (Abcam #ab32).

### Co-immunoprecipitation

Cells were prepared as described above for the GST pull-down assay, 350 µl of supernatant was transferred to co-immunoprecipitation mini-spin columns (Pierce) with HA or Myc antibody (Abcam #ab32) and incubated overnight with gentle rotation at 4 °C. Then 50 µl of a 50% protein G agarose slurry was added to the mixture and further incubated for 5 hrs with rotation. The agarose-protein G/antibody/protein complex was collected by centrifuging at 1300 g for 45∼60 sec. The agarose was washed vigorously 8 times with 400 µl washing buffer (cell lysis buffer plus 0.2% NP40). The protein complex was eluted with 30 µl elution buffer (PIERCE), mixed and centrifuged at 1300 g for 60 sec. The eluant was neutralized immediately by adding 1 µl of 1 M Tris, pH 9.5 per 20 µl of elution buffer. This step was repeated and the two eluates were pooled in one collection tube. Samples were then prepared for analysis by western blot with anti-Myc (Abcam #ab32), and anti-cystin ([38] #70053).

### RT-PCR

The RNeasy Mini Kit (QIAGEN) was used to isolate total RNA from mouse mIMCD-3 cells and the Superscript First-strand Synthesis System (Invitrogen) was used for cDNA synthesis. PCR was then performed with the following primers:

for cystin 5′-GTCCATGAATCCTCAGAACACAACC-3′ (forward); and 5′-CTCAGCCATTCGGTAGACACTC-3′ (reverse); for necdin 5′-GATGCAGATCATGGAGTTCCTGG-3′ (forward) and 5′-AAGTGCCTACACTGAGAACAGTCC-3′ (reverse).

### Immunofluorescence

Cystin::GFP ; necdin::RFP stable transformants were seeded onto transwell tissue culture polyester membrane (Costar 3470 for *enface* imaging) and cultured to 5 days beyond confluence. To block nuclear export of cystin::GFP, stable cells were treated with 80 nM leptomycin B (LMB) for 5 hrs. Cells were washed with PBS, fixed with 4% para-formaldehyde, permeabilized with 0.2% Triton X-100, washed with PBS and counterstained with Hoechst 33258. The membrane was cut out and mounted with ProLong Gold (Molecular Probes) on slides for *enface* imaging of the monolayer. Fluorescently-labeled cells were analyzed on a Leica scanning laser confocal microscope configured with both an Argon Ion (5 mW, 488 nm) and a Krypton Ion (10 mW, 568 nm) laser.

### Luciferase Reporter assay

The core promoter sequence, *Myc* P1 (–88∼+47) was cloned into the firefly luciferase vector pGL4.22. Fresh (<5 passages) mIMCD-3 (ATCC #CRL-2123) cells were seeded in 24-well plates and grown to ∼80–90% confluence, and then transfected with various plasmids using Lipofectamine 2000. All samples represented in Figure 6C were transfected with 15 ng of control Renilla plasmid; lane 1 received 0.3 µg empty vector (pGL4) while lanes 2–7 got 0.3 µg of pGL4.22-*Myc* P1. pcDNA 3.1 was used for filler DNA in combination with pCMV-myc-necdin, pCMV-HA-cystin, and pCMV-HA-cystin-C25 each used at 0.6 µg. Transfected cells were incubated for 48 hrs and the cells were lysed in 100 µl passive lysis buffer (Promega) and shaken for 20 min at room temperature. Firefly and *Renilla* luciferase activities were measured with Dual Luciferase Reporter Assay System (Promega). The luminometer (CENTRO LB 960 Microplate Luminometer, running MikroWin, Version 4.41) was programmed to perform a 1-sec delay with agitation, followed by a 5-sec measurement period for each reporter. 20 µl cell lysate was carefully transferred into 96-well plate, to which 100 µl Luciferase Assay Reagent II was dispensed and luciferase activity was measured, followed by the addition of 100 µl of Stop&Glo and the measurement of Renilla activity. Data are from three independent transfections done in triplicate on three separate occasions.

### Statistical Analysis

In Figure 6C, we calculated the relative luciferase activity in each assay after correcting for transfection differences using the Renilla plasmid. Using triplicate readings from three independent experiments, the data were normalized to lane 2 (pGL4.22-Myc P1) and the SEM was calculated. The 95% confidence interval of the SEM was calculated (SEM x 1.96) and is represented as error bars. All tests were conducted using a 2-tailed type 1 error of 5%.

### Chromatin Immunoprecipitation (ChIP) assay

The ChIP Assay was performed according to the instructions in the ChIP assay kit (Upstate/Milipore). Plasmid DNA of pGL4.22-*Myc* P1 (5 µg), pCMV-myc-necdin (10 µg), pCMV-HA-cystin (10 µg), and pCMV-HA-cystin-C25 (10 µg) in different combinations were transfected with Lipofectamine 2000 (30 µl for 2 µg DNA) into COS-7 cells at 90%–95% confluency. 48 hrs after transfection, the cells were fixed with 1% paraformaldehyde in culture media, and incubated at 37 °C, 5% CO_2_ for 10 min. After fixation, the cells were washed twice with cold 1x PBS buffer containing a protease inhibitor (Sigma, 5 µl/10 ml PBS). The cells were collected and centrifuged at 3000 rpm at 4°C for 5 min. The cell pellet was lysed in 320 µl of SDS lysis buffer plus protease inhibitors, and incubated on ice for 10 min. Sonication was performed with Sonic Dismembrator-60, (power output 6) under the conditions: 10 sec, 10 times with 1 min interval on ice. After sonication, the cell supernatant was collected by centrifuging at 13000 rpm at 4 °C for 5 min and diluted 10-fold in ChIP dilution buffer containing protease inhibitors. Up to 900 µl for each reaction was pre-cleared with ∼30 µl Salmon Sperm DNA/protein A agarose (a 50% slurry) with rotation at 4°C for 1 hr. The agarose was collected at 1000 g for 1min and the supernatant was transferred to a new tube.

The antibodies: anti-cystin, (30 µg/ml, # 70053) or anti-necdin (N143, 10 µg/ml, Abcam #ab18554) were added and the mixtures were incubated overnight at 4°C with constant rotation, followed by the addition and continued incubation with 35 µl Salmon Sperm DNA/protein A for 1.5 hrs with rotation to capture the antibody/protein complex. The protein A agarose-antibody/protein complex was pelleted at 1000 g for 1 min and the supernatant was discarded. The protein A agarose/antibody/protein complex was washed for 12 min each at 4°C with 1 ml low salt buffer, high salt buffer, and finally Lithium chloride wash buffer. Two final washes, each with 1 ml TE buffer were performed at RT. Then, the protein A agarose/antibody/protein complex was re-suspended in 250 µl fresh elution buffer (0.1 M NaHCO3, 1% SDS) and incubated at room temperature for 15 min with rotation. The agarose beads were then spun down and the supernatant was transferred into a new tube. This was repeated and the two eluates (total volume ∼500 µl) were combined. 20 µl 5 M NaCl was added and the mixture was incubated at 65 °C for 4 hrs or overnight to reverse cross-link the DNA. Finally, 10 µl 0.5 M EDTA, 20 µl of 1 M Tris-HCl, pH 6.5 and ∼20 µg proteinase K were added to the tube and incubated at 45°C for 1hr. DNA was recovered with 500 µl phenol/chloroform (phenol:chloroform:IAA, pH 7.9, Ambion) and chloroform:IAA (24:1) extraction. The upper layer was transferred to a new tube. Then precipitation using 1/10 volume 3M sodium acetate (pH 5.2), 40 µg glycogen as a DNA carrier, and a 2.5 volumes ice-cold 100% ethanol was performed after incubation at –70°C overnight. The DNA pellet was collected by centrifuge at 16100 g for 10 min at 4°C. The supernatant was removed and the pellet was washed with 1 ml 70% ethanol and air dried. The DNA was dissolved in 30 µl ddH_2_O. Five µl was used as template for a 25 µl PCR reaction. PCR conditions were: 94°C 3 min, 94°C 30 sec, 62°C 30 sec, 68°C 12 sec, repeat for 33 cycles, 68°C 5 min, 4°C holding. The PCR forward primer was: 5′-CCGCTCGAGGAGAGAGGTGGGGAAGGGAGAAAG-3′ and the reverse primer was: 5′-CCCAAGCTTAGTGAGGCGAGTCGGACCCGGCA-3′.

### Electrophoretic mobility shift assay (EMSA)

mIMCD-3 cells were transfected with pCMV-HA vector, pCMV-cystin-HA, or pCMV-necdin-myc respectively. Nuclear extracts were prepared from mIMCD-3 cells using ProteoExtract Subcellular Proteome Extraction kit (Merck KGaA, Darmstadt, Germany). The gel shift binding reaction was performed according to the instructions of the LightShift Chemiluminescent EMSA kit (Pierce). Binding reagents and conditions were: 200 fmol 5′ biotin-labeled probe (synthesized from Integrated DNA Technologies, Inc.), 4 pmol unlabelled competitor, 2 µl 10X binding reaction buffer, 1 µl 1% NP40, 1 µl 1 M MgCl_2_, 1 µl nuclear extract (1 µg/µl),1 µl dA•dT (1 µg/µl, Sigma) a nonspecific DNA competitor in a total volume of 20 µl. The binding reaction was performed at RT for 20 min, then 5 µl 5X loading buffer was added and mixed well. DNA-protein binding was analyzed by electrophoresis using a 6% retardation gel (Invitrogen) with 0.5X TBE buffer, at 100V for 70 min. After electrophoresis, the DNA-protein complex was transferred to Nylon membrane (pre-soaked in 0.5X TBE for 20 min) using 0.5X TBE (pre-cooled to ∼10°C) buffer. When the transfer was complete, the membrane was placed DNA side up on a dry paper towel and immediately cross-linked for 1-2 minutes using the auto cross-link function of the UV Stratalinker 2400 (Stratagene Inc.). After cross-linking, the biotin-labeled DNA was detected with the Chemiluminescent Nucleic Acid Detection Module (Pierce) following the manufacturer’s instructions.

### Antibodies

Necdin (N20), Santa Cruz, #18255; Nedcin (N143 for ChIP), Abcam, #ab18554; Histone H3, Cell Signaling, #9717; Histone H1, Santa Cruz, #10806; Calpain-2, Cell Signaling, #2539; pCadherin, Santa Cruz, #7893; Cytokeratin cocktail, Oncogene, #CP68; Myc, clone generated at UAB, #9E10; HA, covance, #MMS-101P; c-Myc, Cell Signaling, #5605.
